# Bariatric and metabolic surgery in patients with low body mass index: an online survey of 543 bariatric and metabolic surgeons

**DOI:** 10.1186/s12893-023-02175-4

**Published:** 2023-09-09

**Authors:** Shahab Shahabi Shahmiri, Chetan Parmar, Wah Yang, Panagiotis Lainas, Sjaak Pouwels, Amir Hossein DavarpanahJazi, Sonja Chiappetta, Yosuke Seki, Islam Omar, Ramon Vilallonga, Radwan Kassir, Syed Imran Abbas, Ahmad Bashir, Rishi Singhal, Lilian Kow, Mohammad Kermansaravi

**Affiliations:** 1 Department of Surgery, Minimally Invasive Surgery Research Center, Division of Minimally Invasive and Bariatric Surgery, School of Medicine, Rasool-E Akram Hospital, Iran University of Medical Sciences, Tehran, Iran; 2 Centre of Excellence of European Branch of International Federation for Surgery of Obesity, Hazrat_e Rasool Hospital, Tehran, Iran; 3 Iran National Centre of Excellence for Minimally Invasive Surgery Education, Iran University of Medical Sciences, Tehran, Iran; 4 Consultant Surgeon and Head of Department, Whittington Hospital, London, UK; 5 Department of Metabolic and Bariatric Surgery, the First Affiliated Hospital of Jinan University, Guangzhou, China; 6 Department of Surgery, Metropolitan Hospital, HEAL Academy, Athens, Greece; 7 Department of Minimally Invasive Digestive Surgery, Antoine-Béclère Hospital, Paris-Saclay University, Clamart, France; 8 Department of Intensive Care Medicine, Elisabeth-Tweesteden Hospital, Tilburg, the Netherlands; 9 Obesity and Metabolic Surgery Unit, Ospedale Evangelico Betania, Naples, Italy; 10 Weight Loss and Metabolic Surgery Center, Yotsuya Medical Cube, Tokyo, Japan; 11 Wirral University Teaching Hospital, Birkenhead, UK; 12 Obesity and Metabolic Surgery Unit, Vall Hebron Campus Hospital, Barcelona, Spain; 13 Department of Digestive Surgery, CHU Félix Guyon, Saint Denis, La Réunion, France; 14 Director Obesity & Metabolic Surgery Clinic, Iranian Hospital Dubai, UAE. CEO & Founder of GLR International, Dubai, UAE; 15 GBMC-Jordan Hospital, Amman, Jordan; 16 Consultant Bariatric & Upper GI Surgeon, Birmingham Heartlands Hospital, University Hospital Birmingham, UK. Honorary Senior Lecturer, University of Birmingham, Medical Director, Healthier Weight, Birmingham, UK; 17 Flinders University South Australia, Adelaide, Australia

**Keywords:** Bariatric surgery, Metabolic surgery, Low BMI, Survey

## Abstract

**Background:**

Metabolic and bariatric surgery (MBS) in patients with low body mass index patients is a topic of debate. This study aimed to address all aspects of controversies in these patients by using a worldwide survey.

**Methods:**

An online 35-item questionnaire survey based on existing controversies surrounding MBS in class 1 obesity was created by 17 bariatric surgeons from 10 different countries. Responses were collected and analysed by authors.

**Results:**

A total of 543 bariatric surgeons from 65 countries participated in this survey. 52.29% of participants agreed with the statement that MBS should be offered to class-1 obese patients without any obesity related comorbidities. Most of the respondents (68.43%) believed that MBS surgery should not be offered to patients under the age of 18 with class I obesity. 81.01% of respondents agreed with the statement that surgical interventions should be considered after failure of non-surgical treatments.

**Conclusion:**

This survey demonstrated worldwide variations in metabolic/bariatric surgery in patients with class 1 obesity. Precise analysis of these results is useful for identifying different aspects for future research and consensus building.

## Introduction

Obesity rates are rising globally and simultaneously there is an increase of class I obesity patients, the latter being defined as patients with Body Mass Index (BMI) of ≥ 30 and < 35 kg/m^2^. Patients with class 1 obesity may have similar obesity-related diseases compared to patients with higher grades of severe obesity [1]. Addressing obesity and obesity-related comorbidities can be extremely challenging. Metabolic Bariatric Surgery (MBS) has been proved safe and effective for the management of obesity, its associated comorbidities and metabolic syndrome [2].

The guidelines of eligibility for MBS based on a threshold BMI of 35 kg/m^2^ were adopted back in 1991 [3]. However, recently the National Institute for Health and Care Excellence (NICE) recommended that bariatric surgery should be considered for people with BMI of 30—34.9 kg/m^2^ who have recent-onset Type 2 diabetes mellitus (T2DM) [4]. Various bariatric procedures have been shown to be effective for patients with T2DM in patients who are overweight [5] or suffering from class 1 obesity [6, 7]. There is also some evidence of the role of pharmacotherapy in this low BMI patient group which is shown to be safe and effective when compared to the group of patients with BMI > 35 kg/m^2^ [8]. Similarly, endoscopic interventions like endoscopic sleeve gastroplasty (ESG) have recently shown promising short-term results in low BMI patients [9].

Furthermore, it has been reiterated by the World Health Organization (WHO) in their expert consultation in 2004, the International Diabetes Federation (IDF) position statement in 2011, IFSO-APC consensus statement and NICE guideline that Asians have a higher percentage of body fat than Caucasian people of the same age, sex and BMI. Thus, WHO recommended that for many Asians the limits for public health action should be 23 kg/m^2^. The categories suggested for Asians were: less than 18.5 kg/m^2^ (underweight); 18.5–23 kg/m^2^ (normal); 23–27.5 kg/m^2^ (overweight) and 27.5 kg/m^2^ or higher (obesity). They concluded that a surgical approach may be considered as non-primary alternative to treat inadequately controlled T2DM or metabolic syndrome for suitable Asian candidates with BMI greater than 27.5 kg/m^2^ [4]. Recently the 2022 American Society of Metabolic and Bariatric Surgery (ASMBS) and International Federation for the Surgery of Obesity and Metabolic Disorders (IFSO) guideline recommended BMI thresholds should be adjusted in the Asian population such that a BMI > 25 kg/m^2^ suggests clinical obesity, and individuals with BMI > 27.5 kg/m^2^ should be offered MBS [10].

There are also different guidelines for cut off of BMI eligibility for MBS based on patient ethnicity [4]. As expected, this leads to wide variations from country to country on the practice of MBS. The role of MBS in low BMI group is a recent hot topic of debate. There are also no clear guidelines as to whether MBS should be offered in this patient group without any obesity associated comorbidities. Furthermore, although safety and efficacy of MBS in class 1 obesity has been proven by several studies [11, 12], to date there is paucity of data about appropriate type of surgery, optimal limb length, appropriate size of bougie for sleeve gastrectomy (SG) and perioperative management in this group.

The aim of this global survey is to comprehend opinions of bariatric surgeons around the globe regarding MBS for patients with low BMI. This would provide the opportunity to establish guidelines for this subgroup of patients in the near future.

## Methods and materials

A voluntary, online questionnaire-based survey (https://www.surveymonkey.com/r/LOWBMI) was conducted in the global community of bariatric and metabolic surgeons. The 35 items of the questionnaire were designated by 17 experienced bariatric surgeons from 10 countries based on existing controversies and challenges regarding the perioperative management of patients with BMI under 35 kg/m^2^.

The online questionnaire was anonymous. All methods and procedures used in this study were performed in accordance with the relevant guidelines such as the Declaration of Helsinki and internal guidelines of the Iran University of Medical Sciences (IUMS).

The study protocol was submitted to the ethics committee of the IUMS. The ethics committee has granted an exemption from requiring ethics approval, since the questionnaire was conducted anonymously, and the participating individuals provided their informed consent prior to filling the questionnaire. The participants’ consent was obtained and recorded via the survey application.

The questionnaire was divided into four parts, which included multiple choice questions concerning: definition, indications and contraindications, preoperative investigations, surgical details and postoperative management (see Tables 1, 2, 3 and 4). Respondents could choose more than one answer for some of the questions. To ensure that respondents could also enter other options, a comment box was available at the end of each part of the survey. The survey was made accessible on September 10^th^ 2021 and closed for analysis on December 8^th^ 2021.

**Table 1:** Definition and indications of MBS in patients with low BMI as reported by the participants of the survey

Questions	**Responses** (percentage) the most reported answer was bolded						
What is your lowest BMI cut off for the definition of class I obesity in your country?	**25 kg/m**^**2**^ (6.27%)	**27.5 kg/m**^**2**^ (13.49%)	**30 kg/m**^**2**^** (61.20%)**	**32.5 kg/m**^**2**^ (6.51%)	**35 kg/m**^**2**^ (12.53%)		
What is your lowest BMI cut off for performing MBS in cases do not have obesity related disease?	**25 kg/m** (1.45%)	**27.5 kg/m**^**2**^ (6.51%)	**30 kg/m**^**2**^ (22.89%)	**32.5 kg/m**^**2**^ (13.98%)	**35 kg/m**^**2**^** (26.27%)**	**37.5 kg/m**^**2**^ (3.37%)	**40 kg/m**^**2**^ (25.54%)
What is your lowest BMI cut off for performing MBS in cases who suffer from obesity related disease?	**25 kg/m**^**2**^ (3.13%)	**27.5 kg/m**^**2**^ (18.07%)	**30 kg/m**^**2**^** (33.73%)**	**32.5 kg/m**^**2**^ (13.01%)	**35 kg/m**^**2**^ (31.33%)	**37.5 kg/m**^**2**^ (0.48%)	**40 kg/m** (0.24%)
Do you consider special ethnicities for definition of class 1 obesity?	**Yes** (35.51%)	**NO** **(64.49%)**					
Should bariatric surgery be proposed to patients with class 1 obesity without obesity related comorbidities?	**Yes** **(52.29%)**	**NO** (47.71%)					
Do you offer MBS in cases with class 1 obesity (you can choose more than one answer):	**None of the above** (6.99%)	**Suffering poor control T2DM** **(88.43%)**	**Have other obesity related disease such as HTN, sleep apnea, PCOS and etc** (71.81%)	**Have recently had LAGB removed for complications** (46.02%)	**Have tried dieting off and on for long time** (44.34%)	**Have severe GERD** (47.95%)	
Do you think insurance should consider low BMI for coverage?	**Irrespective of BMI** (15.66%)	**BMI > 30** (20.00%)	**BMI > 30 with comorbidities** **(50.84%)**	**I do not have any idea** (13.49%)			
Would you offer bariatric surgery in cases with class 1 obesity less than 18 years old?	**Yes** (31.57%)	**NO** **(68.43%)**					
Would you consider bariatric surgery in cases with class 1 obesity older than 65?	**Yes** **(55.90%)**	**NO** 44.10%

**Table 2:** Preoperative management of MBS in patients with low BMI as reported by the participants of the survey

Questions	**Responses** (percentage) the most reported answer was bolded	
Should all cases with class 1 obesity undergo preoperative eating disorder and psychological assessment anyway?	**Yes** **(84.56%)**	**NO** (15.44%)
Do you consider nutritional assessment for all cases with class I obesity?	**Yes** **(91.90%)**	**NO** (8.10%)
Do you prefer performing body composition studies preoperatively in this group (for example: fat mass and muscle mass)	**Yes (65.82%)**	**NO** (34.18%)
Should all patients in this group have MDT evaluation?	**Yes** **(82.28%)**	**NO** (17.72%)
Do you consider pharmacotherapy (for example Liraglutide) for this group before recommend to MBS?	**Yes** **(64.81%)**	**NO** (35.19%)
Non-surgical therapies including diet and exercise and pharmacotherapy are effective and durable in long term weight reduction and resolutions of comorbidities for class 1 obesity?	**Agree** (38.99%)	**Disagree** **(61.01%)**
Surgical interventions should be considered just after failure of nonsurgical treatments?	**Agree** **(81.01%)**	**Disagree** (18.99%)

**Table 3:** Surgical management of MBS in patients with low BMI as reported by the participants of the survey

Questions	**Responses** (percentage) the most reported answer was bolded						
Do you recommend intragastric balloon for this group?	**Yes** **(50.83%)**	**No** (49.17%)					
Do you recommend endoscopic intervention for this group?	**NO** **(68.61%)**	**Yes, Endoscopic plication** (7.50%)	**Yes, Endoscopic sleeve gastroplasty** (22.22%)	**Other** (1.67%)			
Is a reversible MBS such as RYGB/OAGB preferred to an irreversible BS such as SG in class I Obesity?	**Yes** (35.00%)	**NO** **(65.00%)**					
Do you think MBS is cost-effective in this group?	**Yes** **(84.72%)**	**NO** (15.28%)					
What should be the procedure of choice for adolescents with class 1 obesity (under 18 years old)? (you can choose more than one answer)	^a^						
What should be the procedure of choice for patients with class 1 obesity in 18 to 65 years old? (you can choose more than one answer)):	^a^						
What should be the procedure of choice for patients with obesity class 1 older than 65 years old? (you can choose more than one answer)	^a^						
What should be the alimentary limb (AL) and Bilio-Pancreatic limb (BPL) length for RYGB in this group? (you can choose more than one answer)	**None of the above** (8.89%)	**BPL of 50cm** (18.89%)	**BPL of 50–100 cm** **(48.89%)**	**BPL of > 100cm** (18.89%)	**AL of 50cm** (6.67%)	**AL of 50-100cm** **(38.61%)**	**AL of > 100cm** (25.83%)
Do you think gastric pouch volume and gastrojejunostomy(GJ)size should be larger than standard to allow eat better to prevent malnutrition in this low BMI group?(you can choose more than one answer)	**None of the above** (11.67%)	**GJ of < 30mm** (29.17%)	**GJ of 30-45mm** **(40.28%)**	**GJ of > 45mm** (6.94%)	**Pouch volume of < 30cc** (10.28%)	**Pouch volume of 30-50cc** **(45.00%)**	**pouch volume of > 50cc** (20.28%)
What should be the BilioPancreatic limb (BPL) length for OAGB in this group?	**100cm** (14.17%)	**120cm** (13.61%)	**150cm** **(33.89%)**	**180cm** (11.39%)	**One third of whole small bowel length** (13.33%)	**Other** (13.61%)	
In class 1 obesity, Laparoscopic Sleeve Gastrectomy should be based on what bougie size?	**32–36 fr** (21.39%)	**36–40 fr** **(63.61%)**	**40–44 fr** (10.28%)	**44–48 fr** (2.22%)	**Other** (2.50%)		
Do you agree that for patients who have lost enough weight after their bypass surgery to reverse to normal anatomy?	**Yes** (11.67%)	**NO** **(88.33%)**					
Is single incision surgery (SILS) suitable and safe for this patients?	**Yes** 41.94%	**NO** **(58.06%)**					

^a^Results illustrated in Fig. 1A, B &C

**Table 4:** Postoperative management of MBS in patients with low BMI as reported by the participants of the survey

Questions	Responses (percentage)) the most reported answer was bolded					
What should be the post-operative follow-ups and para-clinical assessments interval in this group of patients?	**Similar follow up as other groups** **(87.08%)**	**Shorter interval** (10.39%)	**Longer interval** (2.53%)			
How long would you recommend immediate postoperative anti-coagulant duration in this group?	**During hospital stay** **(25.56%)**	**1 week** (24.16%)	**2 weeks** (18.26%)	**3 weeks** (4.78%)	**4 weeks** (11.24%)	**Not routine need (just in selected patients)** (16.01%)
Is postoperative vitamin supplementation recommended for all patients?	**Yes** **(92.70%)**	**NO** (7.30%)				
Which factor may be more accurate for reporting weight loss outcomes in class 1 obesity cases?	**EWL%** **(57.02%)**	**TWL%** (28.09%)	**EBMIL%** (13.48%)			
Ideal body weight in class 1 obesity should be defined based on BMI of…?	**20 kg/m**^**2**^ (10.39%)	**25 kg/m**^**2**^ **(56.46%)**	**30 kg/m**^**2**^ (11.24%)	**I have no comment** (15.17%)		
Weight loss Failure after 18 months of MBS in class I obesity patients is defined as:(You can choose more than one answer)	**None of the above** (1.97%)	**EWL < 25%** (19.66%)	**EWL < 50%** **(51.97%)**	**BMI > 25 kg/m**^**2**^ (12.92%)	**BMI > 30 kg/m**^**2**^ (24.72%)	**weight regain > 10% of best the lowest weight** **achieved** (21.91%)

The survey link was widely shared by authors with surgeons in their network, bariatric and metabolic surgery professional groups and various social media platforms (Facebook™, Researchgate™, Twitter™, WhatsApp™ and LinkedIn™). The email was also sent to known bariatric surgeons who were invited to participate and were also asked to diffuse the survey link to colleagues. Data were analysed using descriptive statistics. Continuous variables were expressed as mean ± standard deviation (SD) and categorical variables as frequency with percentages.

## Results

### General Characteristics

In total, 548 responses were gathered. Among the respondents, 543 (99.09%) were bariatric surgeons and 5 (0.91%) were not. 148(27%) of respondents were from Europe, 123(22%) from Asia, 115(20%) from South America, 104(19%) from Middle East, 28(5%) from Africa and 25(4.5%) from North America. Country of Origin of Respondents was shown in Table 5. In terms of number of metabolic surgeries performed by individual respondents, the following were found: 153 (27.92%) had performed < 200 procedures, 109 (19.89%) between 201 and 500 procedures, 63 (11.50%) between 501 and 1000 procedures and finally 223 (40.69%) more than 1000 procedures.

**Table 5:** Country of Origin of Respondents in Alphabetical Order

Country of Origin	Number of Responses	Percentage
**Algeria**	1	0.18%
**Argentina**	31	5.65%
**Australia**	6	1.09%
**Austria**	1	0.18%
**Azerbaijan**	1	0.18%
**Belgium**	1	0.18%
**Bhutan**	1	0.18%
**Bolivia (Plurinational State of)**	1	0.18%
**Brazil**	21	3.83%
**Côte d'Ivoire**	1	0.18%
**Chile**	6	1.09%
**China**	72	13.11%
**Colombia**	8	1.46%
**Costa Rica**	1	0.18%
**Dominican Republic**	1	0.18%
**Ecuador**	6	1.09%
**Egypt**	17	3.10%
**Finland**	1	0.18%
**France**	18	3.28%
**Germany**	7	1.28%
**Greece**	4	0.73%
**Guatemala**	3	0.55%
**Holy See**	1	0.18%
**Iceland**	1	0.18%
**India**	19	3.46%
Iran** (Islamic Republic of)**	41	7.47%
**Iraq**	3	0.55%
**Israel**	2	0.36%
**Italy**	20	3.64%
**Japan**	25	4.55%
**Jordan**	3	0.55%
**Kuwait**	2	0.36%
**Lebanon**	10	1.82%
**Libya**	3	0.55%
**Malaysia**	2	0.36%
**Mexico**	31	5.65%
**Morocco**	1	0.18%
**Nepal**	1	0.18%
**Netherlands**	7	1.28%
**Nicaragua**	1	0.18%
**Niger**	1	0.18%
**Norway**	2	0.36%
**Pakistan**	18	3.28%
**Palestine State**	1	0.18%
**Panama**	2	0.36%
**Paraguay**	2	0.36%
**Peru**	8	1.46%
**Portugal**	7	1.28%
**Romania**	1	0.18%
**Russian Federation**	2	0.36%
**Saudi Arabia**	4	0.73%
**Singapore**	2	0.36%
**Slovenia**	1	0.18%
**South Africa**	1	0.13%
**Spain**	13	2.37%
**Sweden**	1	0.18%
**Switzerland**	2	0.36%
**Syrian Arab Republic**	3	0.55%
**Thailand**	1	0.18%
**Tunisia**	3	0.55%
**Turkey**	28	5.10%
**Ukraine**	1	0.18%
**United Arab Emirates**	17	3.10%
**United Kingdom**	29	5.28%
**United State of America**	18	3.28%

### Definition and indications

#### BMI cut off points

Regarding the lowest cut off point for considering MBS in patients without any obesity-related comorbidities, 1.45% of participants answered that it should be at a BMI of 25 kg/m^2^, 6.51% at a BMI of 27.5 kg/m^2^, 22.89% at a BMI of 30 kg/m^2^, 13.98% at a BMI of 32.5 kg/m^2^, 26.27% at a BMI of 35 kg/m^2^, 3.37% at a BMI of 37.5 kg/m^2^ and 25.54 at a BMI of 40 kg/m^2^.

For patients that do suffer from obesity-related comorbidities, the lowest BMI cut off distribution was as following: 13 (3.13%) BMI of 25 kg/m^2^; 75 (18.07%) BMI of 27.5 kg/m^2^; 140 (33.73%) BMI 30 kg/m^2^; 54 (13.01%) BMI 32.5 kg/m^2^; 130 (31.33%) BMI 35 kg/m^2^; 2 (0.48%) BMI of 37.5 kg/m^2^ and 1 (0.24%) BMI of 40 kg/m^2^ (data are summarized in Table 1).

#### Other considerations

A total of 147 respondents (35.51%) believed that special ethnicities consideration is necessary for defining class 1 obesity. Two-hundred and seventeen respondents (52.29%) agreed to the fact that MBS should be offered to the class-1 obese patients without any obesity-related comorbidities.

In specific cases, MBS should be offered in patients with class I obesity and poorly controlled T2DM (367 respondents; 88.43%) or patients having other obesity-related comorbidities (298 respondents; 71.81%) (Table 1).

Most respondents (284; 68.43%) answered that MBS should not be proposed to adolescent patients (under 18 years-old) with class I obesity, but it should be considered for patients older than 65 years-old (55.90% of participants' responses).

Most respondents believed that MBS in patients with a BMI > 30 kg/m^2^ with comorbidities should be covered by insurance companies.

#### Preoperative evaluation and specific therapies prior to surgery

A total of 334 respondents (84.56%) agreed that patients with class I obesity should undergo preoperative eating disorder and psychological assessment. Similar agreement was found regarding preoperative nutritional assessment; 363 respondents (91.9%) agreed that this needs to be considered. In addition, 260 (65.82%) respondents agreed that body composition studies need to be performed and a multidisciplinary team (MDT) evaluation is essential (325 respondents, 82.28%).

64.81% respondents recommended pharmacotherapy before MBS. However, 61.01% of respondents disagreed on the fact that non-surgical therapies (including diet and exercise) are effective and durable in long-term weight reduction and resolution of comorbidities for class 1 obesity patients. Therefore, 320 (81.01%) respondents agreed on the fact that surgical interventions should be considered after failure of non-surgical treatments (Table 2).

#### Appropriate bariatric surgical procedure for patients with class I obesity

Regarding intragastric balloon placement, 183 of 360 respondents (50.83%) believed that it can be considered in class I obesity. Endoscopic interventions were not found suitable by 246 of 360 respondents (68.61%). Only a minority of participants considered either endoscopic plication (27 of 360 respondents; 7.50%) or endoscopic sleeve gastroplasty (80 of 360 respondents; 22.22%) in patients with class I obesity. Only 126 of 360 respondents (35.0%) preferred a Roux-en-Y gastric bypass (RYGB) or one anastomosis gastric bypass (OAGB) for patients with class I obesity. Despite variation in responses, most respondents (305/360; 84.72%) believed that metabolic surgery is cost effective in patients with class I obesity.

**Fig. 1:**
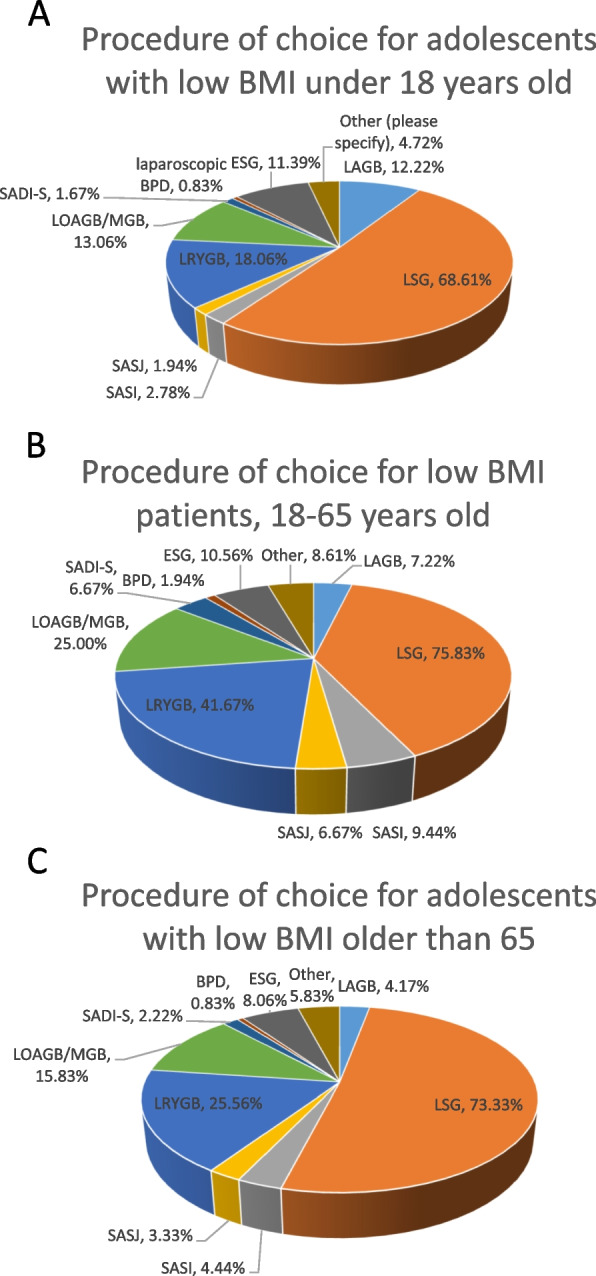
Procedure of choice for three age categories of patients with low BMI, as reported by the participants of the survey. **A**. under 18 years old, **B**.18 to 65 years old, **C**. older than 65 years old. LAGB, Laparoscopic Adjustable Gastric Banding; LSG, Laparoscopic Sleeve Gastrectomy; LRYGB, Laparoscopic Roux en Y Gastric Bypass; LOAGB/MGB, Laparoscopic One Anastomosis Gastric Bypass/Mini gastric bypass; SADI-S, Single anastomosis duodeno-ileostomy with sleeve gastrectomy; BPL-DS, Biliopancreatic diversion with duodenal switch; EGD, endoscopic sleeve gastroplasty

In patients under 18 years-old, 247 of 360 respondents (68.61%) recommended laparoscopic SG as procedure of choice (Fig. 1A).

In patients between 18 and 65 years-old, 273 of 360 respondents (75.83%) recommended laparoscopic SG as the procedure of choice and 150 of 360 respondents (41.67%) recommended laparoscopic RYGB as the procedure of choice in this age group (Fig. 1B).

In patients older than 65 years, 264 of 360 respondents (73.33%) believed that laparoscopic SG is the procedure of choice while 92 of 360 respondents (25.56%) that laparoscopic RYGB should be the procedure of choice in this age group (Fig. 1C).

Respondents could choose more than one answer for these questions; they were asked about their preferred procedures for each age groups and not only for their procedure of choice.

Intraoperative considerations.

##### RYGB

For both the alimentary limb (AL) and the biliopancreatic limb (BPL) a length of 50 to 100 cm was preferred (139/360 respondents (38.61%) and 176/360 respondents (48.89%), respectively). Pouch volume between 30 to 50 cc was preferred by 162 of 360 respondents (45.0%) and the size of gastrojejunostomy (GJ) between 30 to 45 mm was also preferred by 145 of 360 respondents (40.28%).

##### OAGB

The BPL for OAGB was preferred to be 150 cm (122/360 respondents; 33.89%), or 100 cm (51 of 360 respondents; 14.17%).

Sleeve gastrectomy.

A total of 229 of 360 respondents (63.61%) preferred a bougie size between 36–40 French; 77 of 360 respondents (21.39%) recommended a bougie size between 32 and 36 French.

Other perioperative considerations.

Three hundred and eighteen respondents (88.33%) disagreed that patients who lost a significant amount of weight after bypass surgery should be reversed to normal anatomy.

Of the 360 respondents, 209 (58.06%) do not think that single incision surgery (SILS) is suitable and safe for patients with class I obesity.

#### Postoperative considerations

A total of 310 respondents (out of 356; 87.08%) agreed that postoperative follow-up and clinical assessments should be similar as those in other BMI groups. Regarding anticoagulants use, 91 respondents recommended it during hospital stay (25.56%); 86 of them recommended it for one week (24.16%) and 65 respondents recommended it for two weeks postoperative (18.26%).

In 92.70% (330 of 356 respondents) there was agreement on the fact that postoperative vitamin supplementation is recommended for all patients.

In terms of reporting weight loss in patients with class I obesity, 203 of 356 respondents (57.02%) found %EWL an appropriate metric, whereas 100 of 356 respondents (28.09%) preferred %TWL and 48 of 356 respondents (13.48%) found %EBMIL to be more accurate.

Ideal body weight should be defined on BMI of 25 kg/m^2^ according to 201 of 356 respondents (56.46%) in patients with class 1 obesity and weight loss failure should either be defined as < 50% EWL (185 of 356 respondents (51.97%) or < 25% EWL (70 of 356 respondents (19.66%) or weight regain > 10% (compared to the lowest weight achieved) (88 of 356 respondents (24.72%)).

## Discussion

This survey provides interesting information regarding interventional procedures used throughout the world for the management of patients with class 1 obesity. The answers collected clearly demonstrate that endoscopic or surgical approaches for these patients vary according to nationality-continent or surgical team’s preference, with no worldwide trend. The latter is normal, since there is no actual worldwide consensus for this group of patients and practices differ from one continent to another.

Class I obesity is defined as BMI ≥ 30 and < 35 kg/m^2^, so more than 61% of responders agreed with BMI of 30 for class I obesity, however for Asian countries threshold for obesity is set at a lower BMI than western countries (BMI cut-off point for obesity is 30 kg/m^2^ for West, 25 kg/m^2^ for Asia) [13], mainly due to greater proportion of body fat [14]. Despite different body composition between western and Asian populations outlined by some authors [13] and roughly equal worldwide distribution of participants in this survey, approximately two third of responders did not consider specific ethnicity for class I definition.

Former guidelines suggested MBS for patients with BMI more than 40 or more than 35 in presence of obesity-related comorbidities [13, 14]. The new ASMBS/IFSO guidelines recommend MBS for patients with BMI > 35 kg/m^2^ regardless of obesity-associated medical conditions and for patients with BMI of 30–34.9 kg/m^2^ who do not achieve remarkable or persistent weight loss outcomes or improvement of obesity-associated medical problems using nonsurgical methods [10]. Indeed, there is growing evidence that advocates MBS for patients with BMI 30–35 kg/m^2^, especially when it is difficult to control T2DM [15, 16]. Noun et al. showed safety and efficacy of SG in 541 patients with BMI 30–35 kg/m^2^, with a 5-year follow-up of weight loss outcomes and improvement of obesity-associated medical conditions [17]. A randomised control trial study demonstrated significant superiority of MBS in comparison to non-surgical treatments in long-term diabetes improvement and weight loss outcomes in patients with BMI 30 to 35 kg/m^2^ [18]. Another study by Hong et al. confirmed 5-year efficacy and safety of SG in 75 patients with BMI of 30–35 kg/m^2^ [19]. Interestingly, we observed diversity among responders when we asked about lowest BMI cut off for performing MBS in cases without obesity-related diseases. In return, answers about lowest BMI cut off for performing MBS in cases with obesity-related disease was in the line with previous papers [13, 15], and BMI of 30 and 35 were selected by two third of participants. In addition, the most selected obesity related comorbidity by responders which indicated MBS in class I obesity was poor controlled diabetes as outlined by other studies [20].

Regarding age limitation for performing MBS in class I obesity, there was diversity among answers. Most of surgeons disagreed to perform MBS in patients under the age of 18, and they agreed for MBS in patients older than 65 years. This may be reflecting the existence of more acceptable results of MBS in elderly populations [21], compared to scarce and questionable long-term outcomes of MBS for adolescents [22]. Although MBS seems to be safe and effective to treat obesity in adolescents [23, 24], more evidence is needed to support the indication of MBS for adolescents with class I obesity.

More than half of participants suggested insurance coverage of MBS in patients with class I obesity and obesity-related comorbidities. Insurance coverage of MBS varies among countries. In most countries, insurance coverage is limited to individuals with BMI ≥ 40 kg/m^2^ or with BMI ≥ 35 kg/m^2^ and one or more obesity-related conditions, including diabetes [16]. However, recent cost analysis studies suggest that insurance coverage should be further extended to include patients with class I obesity and diabetes to reduce cost-burden of diabetes treatment [25].

Next to the IFSO statement by Busetto et al. (2014), MBS is indicated in patients with class I obesity suffering from a significant obesity-related health burden and not achieving weight control with nonsurgical therapy in the long term. Therefore, preoperative assessment should focus on obesity-related comorbidities and validation of a reasonable period of nonsurgical therapy [12]. Since BMI level alone is an inaccurate index of adiposity and a poor health risk predictor [26], more than half of this expert group recommend, that body composition studies should be performed preoperatively to identify patient’s global health and a prediction of its future disease risk.

Multidisciplinary approach, preoperative nutritional and psychological assessments were recommended by most of the experts. These responses on consensus underline the importance of the multidisciplinary approach combined with surgical therapy. It is fundamental to remember that RCTs demonstrated that lifestyle modification programs alone may achieve a modest weight loss of 5–7% but only in approximately half of the patients [12]. There is evidence and guidelines to support the role of perioperative non-surgical supports to have better outcomes after MBS [27, 28].

Since pharmacotherapy of obesity is rapidly evolving and new drugs seem to promise important weight loss [29] and improvement of comorbidities [30, 31], most experts in this survey recommend pharmacotherapy prior to surgery. Indeed, the SURMOUNT program including four global trials, showed efficacy of Tirzepatide as a glucagon-like peptide-1 receptor agonist in weight loss in patients with obesity [32]. A recently published review by Pedrosa et al. suggested the efficacy of glucagon-like peptide-1 receptor agonists (GLP-1RA) to treat obesity [33]. Nevertheless, most of the expert group in this survey disagree that nonsurgical therapies are effective and durable in the long term. Finally, most of the expert group consider MBS after failure of nonsurgical treatments.

Endoscopic therapies have emerged over the years, intending to fill the gap between medical and surgical therapies to combat the obesity epidemic, and are particularly used for class 1 obesity patients. Short-term devices such as intragastric balloons have been used in class 1 obesity patients, providing only short-term weight loss with immediate weight regain after their removal [34–36]. Interestingly, half of the surgeons answering this survey recommend intragastric balloon for class 1 obesity patients, reflecting once more the worldwide disparity of approaches for this group of patients. Furthermore, 2/3 of the participants do not recommend any endoscopic procedure for these patients, confirming that more than half of the participants do not include in their arsenal against class 1 obesity any endoscopic procedure. However, 22% of responders would perform an endoscopic sleeve gastroplasty to these patients, a novel procedure that is progressively emerging in the US and in some European and Asian countries with promising weight loss and comorbidities resolution results for class 1 obesity patients in short term [37–39].

Bariatric surgery is now established as the most effective treatment for severe obesity and obesity-related comorbidities [40]. However, when it comes to class 1 obesity patients, data on surgical procedures are scarce and great discrepancies exist between studies. The lack of consensus on the use of bariatric surgery for this group of patients makes the outcomes of this survey even more interesting. Most of participants believe that MBS is cost-effective in class 1 obesity patients, in accordance to what has been previously reported in the literature mainly for patients of this group with T2DM [41, 42]. Future data are necessary to assess whether this stands for all patients with class 1 obesity. When it comes to technique, most participants do not recommend a reversible bariatric procedure (RYGB or OAGB [43]) for these patients. Additionally, laparoscopic SG is preferred by most participating surgeons for all age subgroups (including adolescents) of class 1 obesity patients, corresponding to the existing worldwide metabolic surgery trend for patients with severe obesity. Indeed, nowadays, SG has been established as a widely accepted stand-alone bariatric operation gaining popularity and acceptance among surgeons and is currently the most frequently performed procedure worldwide [40, 44]. It is currently considered simpler and less invasive that the gastric bypass, since it does not require a gastrointestinal anastomosis. In addition, reports in the literature on the success and excellent weight loss after laparoscopic SG have been accumulating over the past few years in all age subgroups [45–48]. Therefore, the preference of survey participants for laparoscopic SG in class 1 obesity patients seems rational when one considers the combination of good weight loss/comorbidities resolution results and the less invasive aspect of laparoscopic SG when compared to other MBS procedures.

The second preferred MBS for class 1 obesity patients in this survey was RYGB, which is in accordance with the current practice in class 2 or 3 obese patients worldwide [40]. Almost half of the participants opted for a standard biliopancreatic limb of 50–100 cm. However, most participants preferred a 50–100 cm alimentary limb for these patients, instead of the 150-cm alimentary limb of the usually performed RYGB. It has been shown that a longer biliopancreatic limb leads to more weight loss after RYGB [49, 50] especially in mid-term follow-ups [51]. Responses are also surprising regarding gastric pouch volume and gastrojejunostomy size. These controversial results reflect once more the lack of common surgical strategies and the urgent need for consensus for patients with class 1 obesity. Finally, most of participants do not advise reversal of the bypass to normal anatomy after sufficient weight loss in these patients, in line with the current practice in severely obese patients, since revisional procedure is associated with high morbidity, including sepsis, leaks and bleeding, high reoperative rates and increased readmission [52].

Interestingly, 42% of participants consider single-incision laparoscopic surgery (SILS) suitable for class 1 obesity patients. This is probably because low BMI patients seem easier to operate on due to less thick abdominal walls and decreased intraabdominal fat. Indeed, for severely obese patients, a recent systematic review suggested that intraoperative results and clinical outcomes of SILS SG, SILS RYGB and SILS gastric banding appear to be comparable with those of conventional laparoscopy [53], while SILS for SG has been proven to be safe and feasible with good outcomes [54]. However, caution should be taken for SILS RYGB, since additional trocars are very often necessary to achieve triangulation and further studies are necessary to draw conclusions on the safety and effectiveness of this technique.

There is no doubt that most respondents believe that postoperative follow-up and para-clinical assessments interval in patients with class I obesity should be similar to other groups. Most respondents agreed that postoperative vitamin supplementation should be recommended for all patients. This is a general practice and in line with suggested guidelines [43, 55]. Inadequate vitamin supplementation may also lead to numerous nutritional deficiencies like hair loss [56, 57]. Therefore, postoperative vitamin supplementation seems essential for most patients after bariatric surgery [43, 58].

Most respondents believe that EWL% is most accurate for reporting weight loss outcomes in class 1 obesity cases. But there was a recommendation that “percent excess BMI loss (%EBMIL) is determined from easily available clinical data, is readily reproducible, and is consistent with other bariatric metrics that rely on BMI, such as obesity classification and thresholds for surgery, making it clinically relevant” [59]. Most respondents agreed that weight loss failure after 18 months of MBS in class I obesity patients is defined as EWL < 50%. However, should we only focus on weight loss? The improvement of obesity associated medical problems after MBS is also important for patients with class 1 obesity and should be factored in when labelling outcomes.

Most respondents believe that ideal body weight in class 1 obesity should be defined based on BMI of 25 kg/m^2^. However, BMI of 25 kg/m^2^ in Asians means overweight. It should be reduced by 2.5 kg/m^2^ for the Asian population just like its BMI criteria for bariatric surgery [60]. New ASMBS/IFSO guideline recommends adjusting BMI 25–27.5 kg/m^2^ as definition of obesity in Asian population [10].

Even if this study provides interesting information regarding the interventional management of patients with class 1 obesity worldwide, it has several limitations. The main limitation is that even though IFSO currently counts more than 6500 members with the clear majority of them being bariatric surgeons, only 543 participated in this survey. Subsequently, a minority of members have responded to our questionnaire. Additionally, important variances exist on worldwide practices for class I obesity patients, making it difficult to depict all of them through this questionnaire. However, to date, this is the largest survey addressing MBS practices in patients with low body mass index.

## Conclusion

This international survey highlights worldwide variations in practices for patients with low BMI. There is a need for international consensus on developing uniformity in managing this group of patients.

## Data Availability

The data that support the result of this study are available on request from the corresponding author. The data are not publicly available due to privacy or ethical restrictions.

## References

[1] Trends in adult body-mass index in 200 countries from 1975 to 2014: a pooled analysis of 1698 population-based measurement studies with 19·2 million participants. Lancet. 2016;387(10026):1377-96. 10.1016/S0140-6736(16)30054-X PMC7615134 27115820

[2] Schauer PR, Bhatt DL, Kirwan JP, Wolski K, Aminian A, Brethauer SA (2017). Bariatric Surgery versus Intensive Medical Therapy for Diabetes - 5-Year Outcomes. N Engl J Med.

[3] Gastrointestinal surgery for severe obesity (1992). National Institutes of Health Consensus Development Conference Statement. Am J Clin Nutr.

[4] Stegenga H, Haines A, Jones K, Wilding J. Identification, assessment, and management of overweight and obesity: summary of updated NICE guidance. Bmj. 2014;349. 10.1136/bmj.g6608 25430558

[5] Huang Z-P, Guo Y, Liu C-Q, Qi L, Zou D-J, Zhou W-P (2018). The effect of metabolic surgery on nonobese patients (BMI< 30 kg/m2) with type 2 diabetes: a systematic review. Surgery for Obesity and Related Diseases.

[6] Reis CE, Alvarez-Leite JI, Bressan J, Alfenas RC (2012). Role of bariatric-metabolic surgery in the treatment of obese type 2 diabetes with body mass index< 35 kg/m2: a literature review. Diabetes Technol Ther.

[7] Parmar CD, Zakeri R, Mahawar K (2020). A systematic review of one anastomosis/mini gastric bypass as a metabolic operation for patients with body mass index≤ 35 kg/m 2. Obes Surg.

[8] le Roux C, Aroda V, Hemmingsson J, Cancino AP, Christensen R, Pi-Sunyer X. Comparison of Efficacy and Safety of Liraglutide 3.0 mg in Individuals with BMI above and below 35 kg/m^2^: A Post-hoc Analysis. Obesity facts. 2017;10(6):531–44. 10.1159/000478099 PMC5836203 29145215

[9] Yoon JY, Arau RT (2021). The efficacy and safety of endoscopic sleeve gastroplasty as an alternative to laparoscopic sleeve gastrectomy. Clinical Endoscopy.

[10] Eisenberg D, Shikora SA, Aarts E, Aminian A, Angrisani L, Cohen RV (2022). 2022 American Society for Metabolic and Bariatric Surgery (ASMBS) and International Federation for the Surgery of Obesity and Metabolic Disorders (IFSO): Indications for Metabolic and Bariatric Surgery. Surg Obes Relat Dis.

[11] Feng X, Andalib A, Brethauer SA, Schauer PR, Aminian A (2019). How safe is bariatric surgery in patients with class I obesity (body mass index 30–35 kg/m(2))?. Surg Obes Relat Dis.

[12] Busetto L, Dixon J, De Luca M, Shikora S, Pories W, Angrisani L (2014). Bariatric surgery in class I obesity : a Position Statement from the International Federation for the Surgery of Obesity and Metabolic Disorders (IFSO). Obes Surg.

[13] An S, Park HY, Oh SH, Heo Y, Park S, Jeon SM (2020). Cost-effectiveness of Bariatric Surgery for People with Morbid Obesity in South Korea. Obes Surg.

[14] Gallagher D, Heymsfield SB, Heo M, Jebb SA, Murgatroyd PR, Sakamoto Y (2000). Healthy percentage body fat ranges: an approach for developing guidelines based on body mass index. Am J Clin Nutr.

[15] Worldwide trends in body-mass index (2017). underweight, overweight, and obesity from 1975 to 2016: a pooled analysis of 2416 population-based measurement studies in 128·9 million children, adolescents, and adults. Lancet.

[16] Rubino F, Nathan DM, Eckel RH, Schauer PR, Alberti KG, Zimmet PZ (2016). Metabolic Surgery in the Treatment Algorithm for Type 2 Diabetes: A Joint Statement by International Diabetes Organizations. Surg Obes Relat Dis.

[17] Noun R, Slim R, Nasr M, Chakhtoura G, Gharios J, Antoun NA (2016). Results of Laparoscopic Sleeve Gastrectomy in 541 Consecutive Patients with Low Baseline Body Mass Index (30–35 kg/m(2)). Obes Surg.

[18] O'Brien PE, Brennan L, Laurie C, Brown W (2013). Intensive medical weight loss or laparoscopic adjustable gastric banding in the treatment of mild to moderate obesity: long-term follow-up of a prospective randomised trial. Obes Surg.

[19] Hong JS, Kim WW, Han SM (2015). Five-year results of laparoscopic sleeve gastrectomy in Korean patients with lower body mass index (30–35 kg/m^2^). Obes Surg.

[20] Ke Z, Li F, Gao Y, Tan D, Sun F, Zhou X (2021). The Use of Visceral Adiposity Index to Predict Diabetes Remission in Low BMI Chinese Patients After Bariatric Surgery. Obes Surg.

[21] Vallois A, Menahem B, Alves A (2020). Is Laparoscopic Bariatric Surgery Safe and Effective in Patients over 60 Years of Age?" an Updated Systematic Review and Meta-Analysis. Obes Surg.

[22] Shoar S, Mahmoudzadeh H, Naderan M, Bagheri-Hariri S, Wong C, Parizi AS (2017). Long-Term Outcome of Bariatric Surgery in Morbidly Obese Adolescents: a Systematic Review and Meta-Analysis of 950 Patients with a Minimum of 3 years Follow-Up. Obes Surg.

[23] Beamish AJ, Harper ER, Järvholm K, Janson A, Olbers T. Long-term outcomes following adolescent metabolic and bariatric surgery. The Journal of clinical endocrinology and metabolism. 2023. 10.1210/clinem/dgad155 PMC10438888 36947630

[24] Olbers T, Beamish AJ, Gronowitz E, Flodmark CE, Dahlgren J, Bruze G (2017). Laparoscopic Roux-en-Y gastric bypass in adolescents with severe obesity (AMOS): a prospective, 5-year, Swedish nationwide study. Lancet Diabetes Endocrinol.

[25] Hoerger TJ (2019). Economics and Policy in Bariatric Surgery. Curr Diab Rep.

[26] De Luca M, Angrisani L, Himpens J, Busetto L, Scopinaro N, Weiner R (2016). Indications for Surgery for Obesity and Weight-Related Diseases: Position Statements from the International Federation for the Surgery of Obesity and Metabolic Disorders (IFSO). Obes Surg.

[27] Mechanick JI, Apovian C, Brethauer S, Timothy Garvey W, Joffe AM, Kim J (2020). Clinical Practice Guidelines for the Perioperative Nutrition, Metabolic, and Nonsurgical Support of Patients Undergoing Bariatric Procedures - 2019 Update: Cosponsored by American Association of Clinical Endocrinologists/American College of Endocrinology, The Obesity Society, American Society for Metabolic and Bariatric Surgery, Obesity Medicine Association, and American Society of Anesthesiologists. Obesity (Silver Spring, Md).

[28] Parrott J, Frank L, Rabena R, Craggs-Dino L, Isom KA, Greiman L (2017). American Society for Metabolic and Bariatric Surgery Integrated Health Nutritional Guidelines for the Surgical Weight Loss Patient 2016 Update: Micronutrients. Surg Obes Relat Dis.

[29] Friedrichsen M, Breitschaft A, Tadayon S, Wizert A, Skovgaard D (2021). The effect of semaglutide 2.4 mg once weekly on energy intake, appetite, control of eating, and gastric emptying in adults with obesity. Diabetes Obes Metab.

[30] Newsome PN, Buchholtz K, Cusi K, Linder M, Okanoue T, Ratziu V (2021). A Placebo-Controlled Trial of Subcutaneous Semaglutide in Nonalcoholic Steatohepatitis. N Engl J Med.

[31] Li J, He K, Ge J, Li C, Jing Z (2021). Efficacy and safety of the glucagon-like peptide-1 receptor agonist oral semaglutide in patients with type 2 diabetes mellitus: A systematic review and meta-analysis. Diabetes Res Clin Pract.

[32] le Roux CW, Zhang S, Aronne LJ, Kushner RF, Chao AM, Machineni S (2023). Tirzepatide for the treatment of obesity: Rationale and design of the SURMOUNT clinical development program. Obesity (Silver Spring, Md).

[33] Pedrosa MR, Franco DR, Gieremek HW, Vidal CM, Bronzeri F, de Cassia RA (2022). GLP-1 Agonist to Treat Obesity and Prevent Cardiovascular Disease: What Have We Achieved so Far?. Curr Atheroscler Rep.

[34] Ponce J, Woodman G, Swain J, Wilson E, English W, Ikramuddin S (2015). The REDUCE pivotal trial: a prospective, randomized controlled pivotal trial of a dual intragastric balloon for the treatment of obesity. Surg Obes Relat Dis.

[35] Kotzampassi K, Grosomanidis V, Papakostas P, Penna S, Eleftheriadis E (2012). 500 intragastric balloons: what happens 5 years thereafter?. Obes Surg.

[36] Chan DL, Cruz JR, Mui WL, Wong SKH, Ng EKW (2021). Outcomes with Intra-gastric Balloon Therapy in BMI < 35 Non-morbid Obesity: 10-Year Follow-Up Study of an RCT. Obes Surg.

[37] Singh S, de Moura DTH, Khan A, Bilal M, Chowdhry M, Ryan MB (2020). Intragastric Balloon Versus Endoscopic Sleeve Gastroplasty for the Treatment of Obesity: a Systematic Review and Meta-analysis. Obes Surg.

[38] Cheskin LJ, Hill C, Adam A, Fayad L, Dunlap M, Badurdeen D (2020). Endoscopic sleeve gastroplasty versus high-intensity diet and lifestyle therapy: a case-matched study. Gastrointest Endosc.

[39] Fiorillo C, Quero G, Vix M, Guerriero L, Pizzicannella M, Lapergola A (2020). 6-Month Gastrointestinal Quality of Life (QoL) Results after Endoscopic Sleeve Gastroplasty and Laparoscopic Sleeve Gastrectomy: A Propensity Score Analysis. Obes Surg.

[40] Angrisani L, Santonicola A, Iovino P, Vitiello A, Higa K, Himpens J (2018). IFSO Worldwide Survey 2016: Primary, Endoluminal, and Revisional Procedures. Obes Surg.

[41] Keating CL, Dixon JB, Moodie ML, Peeters A, Playfair J, O'Brien PE (2009). Cost-efficacy of surgically induced weight loss for the management of type 2 diabetes: a randomized controlled trial. Diabetes Care.

[42] Picot J, Jones J, Colquitt JL, Loveman E, Clegg AJ (2012). Weight loss surgery for mild to moderate obesity: a systematic review and economic evaluation. Obes Surg.

[43] Kermansaravi M, Shahmiri SS, Davarpanah Jazi AH, Valizadeh R, Weiner RA, Chiappetta S (2021). Reversal to normal anatomy after one-anastomosis/mini gastric bypass, indications and results: a systematic review and meta-analysis. Surg Obes Relat Dis.

[44] van Rutte PW, Smulders JF, de Zoete JP, Nienhuijs SW (2014). Outcome of sleeve gastrectomy as a primary bariatric procedure. Br J Surg.

[45] Castagneto Gissey L, Casella Mariolo JR, Genco A, Troisi A, Basso N, Casella G (2018). 10-year follow-up after laparoscopic sleeve gastrectomy: Outcomes in a monocentric series. Surg Obes Relat Dis.

[46] Goldberg I, Yang J, Nie L, Bates AT, Docimo S, Pryor AD (2019). Safety of bariatric surgery in patients older than 65 years. Surg Obes Relat Dis.

[47] Lainas P, De Filippo G, Di Giuro G, Mikhael R, Bougneres P, Dagher I (2020). Laparoscopic Sleeve Gastrectomy for Adolescents Under 18 Years Old with Severe Obesity. Obes Surg.

[48] Howard R, Chao GF, Yang J, Thumma J, Chhabra K, Arterburn DE (2021). Comparative Safety of Sleeve Gastrectomy and Gastric Bypass Up to 5 Years After Surgery in Patients With Severe Obesity. JAMA Surg.

[49] Zerrweck C, Herrera A, Sepúlveda EM, Rodríguez FM, Guilbert L (2021). Long versus short biliopancreatic limb in Roux-en-Y gastric bypass: short-term results of a randomized clinical trial. Surg Obes Relat Dis.

[50] Zorrilla-Nunez LF, Campbell A, Giambartolomei G, Lo Menzo E, Szomstein S, Rosenthal RJ (2019). The importance of the biliopancreatic limb length in gastric bypass: A systematic review. Surg Obes Relat Dis.

[51] Darabi S, Pazouki A, Hosseini-Baharanchi FS, Kabir A, Kermansaravi M (2020). The role of alimentary and biliopancreatic limb length in outcomes of Roux-en-Y gastric bypass. Wideochir Inne Tech Maloinwazyjne.

[52] Ma P, Ghiassi S, Lloyd A, Haddad A, Boone K, DeMaria E (2019). Reversal of Roux en Y gastric bypass: largest single institution experience. Surg Obes Relat Dis.

[53] Lainas P, Derienne J, Dammaro C, Schoucair N, Devaquet N, Dagher I (2020). Single-port Laparoscopic Surgery for the Treatment of Severe Obesity: Review and Perspectives. Obes Surg.

[54] Lainas P, Derienne J, Zervaki S, Del Basso C, Malerba V, Devaquet N (2021). Left Hypochondrium or Transumbilical Single-Incision Laparoscopic Sleeve Gastrectomy for the Treatment of Severe Obesity: Surgical Technique and Results of a Tertiary Referral Bariatric Center. Obes Surg.

[55] O'Kane M, Parretti HM, Pinkney J, Welbourn R, Hughes CA, Mok J (2020). British Obesity and Metabolic Surgery Society Guidelines on perioperative and postoperative biochemical monitoring and micronutrient replacement for patients undergoing bariatric surgery-2020 update. Obes Rev.

[56] Hosseini-Esfahani F, Khalaj A, Valizadeh M, Azizi F, Barzin M, Mirmiran P (2021). Nutrient Intake and Deficiency of Patients 1 Year After Bariatric Surgery: Tehran Obesity Treatment Study (TOTS). J Gastrointest Surg.

[57] Zhang W, Fan M, Wang C, Mahawar K, Parmar C, Chen W (2021). Hair Loss After Metabolic and Bariatric Surgery: a Systematic Review and Meta-analysis. Obes Surg.

[58] Zhang W, Fan M, Wang C, Mahawar K, Parmar C, Chen W (2021). Importance of Maintaining Zinc and Copper Supplement Dosage Ratio After Metabolic and Bariatric Surgery. Obes Surg.

[59] Brethauer SA, Kim J, El Chaar M, Papasavas P, Eisenberg D, Rogers A (2015). Standardized outcomes reporting in metabolic and bariatric surgery. Obes Surg.

[60] Yang W, Wang C (2022). Metabolic Surgery Needs Stronger Endorsement in Asian T2DM Patients with Low BMI. Obes Surg.

